# Does Symptom Linkage Density Predict Outcomes in Cognitive Therapy for Recurrent Depression?

**DOI:** 10.1007/s10862-021-09914-y

**Published:** 2021-08-17

**Authors:** Jeffrey R. Vittengl, Lee Anna Clark, Michael E. Thase, Robin B. Jarrett

**Affiliations:** Department of Psychology, Truman State University, Kirksville, Missouri, USA; Department of Psychology, University of Notre Dame, Notre Dame, Indiana, USA; Department of Psychiatry, Perelman School of Medicine, University of Pennsylvania, Philadelphia, Pennsylvania, USA; Department of Psychiatry, The University of Texas Southwestern Medical Center, Dallas, Texas, USA

**Keywords:** depression, cognitive therapy, response, follow-up, symptom linkage density

## Abstract

**Background:**

Acute-phase cognitive therapy (CT) is an efficacious treatment for major depressive disorder (MDD), but how CT helps patients is incompletely understood. As a potential means to clarify CT mechanisms, we defined “symptom linkage density” (SLD) as a patient’s mean time-lagged correlation among nine depressive symptoms across 13 weekly assessments. We hypothesized that patients with higher SLD during CT have better outcomes (treatment response, and fewer symptoms after response), and we explored whether SLD correlated with other possible CT processes (growth in social adjustment and CT skills).

**Method:**

Data were drawn from two clinical trials of CT for adult outpatients with recurrent MDD (primary sample *n* = 475, replication sample *n* = 146). In both samples, patients and clinicians completed measures of depressive symptoms and social adjustment repeatedly during CT. In the primary sample, patients and cognitive therapists rated patients’ CT skills. After CT, responders were assessed for 32 (primary sample) or 24 (replication sample) additional months to measure long-term depression outcomes.

**Results:**

Higher SLD predicted increases in social adjustment (both samples) and CT skills (primary sample) during CT, CT response (both samples), and lower MDD severity for at least 2 years after CT response (both samples). Analyses controlled patient-level symptom means and variability to estimate SLD’s incremental predictive validity.

**Conclusions:**

These novel findings from two independent samples with longitudinal follow-up require further replication and extension. SLD may reflect or facilitate generalization of CT skills, improvement in social functioning, or other processes responsible for CT’s shorter and longer term benefits.

Acute-phase cognitive therapy (CT) is an efficacious treatment for MDD, with outcomes similar to antidepressant medication (Cuijpers et al., 2013). Even so, about one-third to one-half of patients with MDD do not respond to CT (Cuijpers et al., 2014), and the mechanisms by which CT helps (or fails to help) individual patients are not well understood (Kazdin, 2009; Longmore & Worrell, 2007; Vittengl & Jarrett, 2014). Because MDD is defined and diagnosed as a multi-symptom syndrome (American Psychiatric Association, 2013), CT mechanisms may facilitate propagation of initial improvements across a patient’s network of depression symptoms (e.g., better sleep leads to improved concentration). In this context, we investigated a new parameter—the strength of interconnections among depressive symptoms across time—which we term “symptom linkage density” (SLD). Previously proposed mechanisms include patients’ use of skills taught in CT and improvement in psychosocial functioning (Beck & Haigh, 2014; Beck & Bredemeier, 2016; Jarrett et al., 2018). We tested whether higher SLD predicts favorable treatment outcomes in CT for recurrent MDD, and we explored whether SLD correlates with improved psychosocial functioning and CT skills.

Relatively few empirical predictors are available to help forecast—or elucidate the mechanisms influencing—depressed patients’ treatment outcomes (Vittengl & Jarrett, 2014). Better understanding of change processes during treatment that signal a better prognosis holds potential to improve both treatment and treatment selection for patients (Kazdin, 2009; Kraemer, 2016). Drawing from learning, psychopathology, and psychosocial theories, we tested the possibility that patients with better outcomes (vs. their counterparts with poorer outcomes) differ in the average level of interconnection among depressive symptoms over time during CT. This hypothesis grows from the recognition that the underlying structure of depression is a syndrome (as conceptualized in psychopathology) and also evidences response generalization (as conceptualized in learning theory). In other words, depression consists of a set of interconnected symptoms, and CT patients learn new skills that, when applied and practiced, can spread to many symptoms and settings across time. As an example of SLD, Figure 1 depicts time-lagged correlations among depression symptoms. If a patient has a low level of symptom *A* at time *t*, this relative strength may be generalized as a lower level of symptom *B* at time *t*+*1*. In this paper, we operationalized SLD as the average of such time-lagged, within-patient associations among nine core MDD symptoms across 13 weekly assessments (American Psychiatric Association, 2013).

We scored SLD from patient-level network analyses estimating individual differences in symptom intercorrelations from week to week during CT (cf. Bringmann et al., 2013; Epskamp et al., 2018; Pe et al., 2015). A SLD score is descriptive of an individual patient and captures the mean time-lagged correlation among the patient’s nine depressive symptom scores measured for 13 weeks (i.e., 12 one-week intervals with individual symptom increases and/or decreases). For example, a positive SLD value would suggest that lower scores on particular symptoms (e.g., less anhedonia, fewer sleep problems) tend to predict lower scores on other symptoms during the subsequent week (e.g., less suicidality, less guilt). Our descriptive, patient-level use of network analysis facilitates the current study’s hypothesis tests and differs from inferential, nomothetic network analysis that aims to draw conclusions about a population (e.g., which symptoms are most central to a disorder; Forbes et al., 2017; Lorimer et at., 2019).

Past research using patient-level network analysis provided context for the current study. For example, Bringmann et al. (2013) combined idiographic (within-person) and nomothetic (between-person) information in multilevel vector autoregression network analyses to study mood dimensions’ interrelations over time. Participants were 129 adults with residual depressive symptoms who provided mood ratings on up to 60 occasions both before and after mindfulness psychotherapy or wait-list control. Bringmann et al. found that mood dimensions were reinforcing within (e.g., negative mood ratings correlated directly with other negative mood ratings) but inhibiting between (e.g., positive mood ratings correlated inversely with negative mood ratings) domains. However, participating in mindfulness psychotherapy had no clear relation to mood network organization.

Our use of network analysis to score SLD was similar to that of Pe et al. (2015), who compared 53 adults diagnosed with MDD with 53 healthy controls. Participants in the Pe et al. study rated seven negative and four positive mood descriptors on up to 56 occasions. Pe et al. estimated network density as the average strength of time-lagged, within-person relations among mood ratings. The person-level network density estimates were then used to test hypotheses. Pe et al. found that persons with MDD versus healthy controls had denser negative, but not positive, mood networks. Thus, depression may be characterized in part by a self-reinforcing negative affective system.

Similar to Bringmann et al.’s (2013) and Pe et al.’s (2015) findings, we expected patients’ MDD symptoms to be positively interrelated over time (i.e., mean SLD > 0). Extending this literature, we used patients’ SLD scores to test distinct predictions in a different context. We measured SLD during acute-phase CT for MDD, during which large improvements in mood are common. Although CT often begins with behavioral activation (to reengage with sources of reinforcement in the environment) followed by cognitive restructuring (to reduce negatively biased information processing and attendant affect), the specific depression symptoms and life situations addressed in these stages of CT are applied in a patient-specific manner (Beck et al., 1979). Cognitive therapists individualize treatment by collaborating with a patient to identify particularly troublesome target symptoms for which CT skills are learned, applied, and ideally then generalized by the patient to other related symptoms and/or settings over time. In this context, we expected higher SLD scores to predict better short- and long-term CT outcomes.

We also explored whether higher SLD scores related to possible treatment processes during CT. First, patients’ learning and application of skills may be a central mechanism in CT for depression (Beck & Haigh, 2014; Hundt et al. 2012; Jarrett et al., 2018). In the cognitive theory of depression, depressive mood and behavior result from negatively biased information processing activated by perceptions of loss. For example, an adult experiencing a lateral move at work rather than a promotion might perceive this as loss of social status and financial opportunity. These perceptions may activate thoughts (e.g., “I’m incompetent and can’t advance, and everybody knows it.”) that then increase negative emotion (e.g., anxiety, irritability), decrease positive emotion (e.g., optimism, energy), and produce unhelpful behavior (e.g., missing work, avoiding colleagues).

To counter such depressogenic processes, CT patients learn, practice, and apply specific skills to recognize and change relations among emotion, thought, and behavior to reduce depressive symptoms (Jarrett et al., 2011; Strunk et al., 2007). For example, CT patients often test the validity of thoughts, evaluate unrealistically negative thoughts while considering the consequences of relying on more accurate and helpful perspectives, engage in problem solving, and participate in activities correlated with pleasure and positive outcomes. Ideally, such learning and practice facilitate generalization of CT skills across time, context, and symptoms (Jarrett, Vittengl, & Clark 2008). A patient’s successful application of CT skills to one symptom (e.g.,antecedent control to improve sleep) may be reinforcing and thus followed by application of the same or other skills to additional symptoms (e.g., activity scheduling to increase pleasure/interest). From this perspective, we explored whether growth in patients’ CT skills related to higher SLD.

Second, psychosocial functioning may also be important in the onset, persistence, and treatment of depression. Here we define psychosocial functioning as the degree of competence and satisfaction in major life domains, such as work, leisure, and social relationships (Weissman et al., 2001). Reduction of psychosocial functioning via negative life events (e.g., job loss, divorce) is a well-established risk for MDD onset (Colman & Ataullahjan, 2010). In addition, psychosocial functioning improves substantively during efficacious treatment of MDD, whereas residual or recurring psychosocial impairment is a risk factor for depressive relapse (Vittengl & Jarrett, 2014).

Through processes including behavioral activation, patients receiving CT may act to reduce depressive symptoms and improve psychosocial functioning (Kaiser, Hubley, & Dimidjian, 2016). As CT patients reengage with sources of reinforcement, they may experience mutually reinforcing improvements in mood and functioning. For example, CT patients who return to work may experience decreased guilt and improved sleep that, in turn, facilitate reengaging in social activities, leading to increased interest and pleasure. Thus, improving psychosocial functioning during CT may help spread reductions across a network of depressive symptoms, as reflected in higher SLD. Accordingly, we explored the correlation of SLD with CT patients’ psychosocial functioning in the current analyses.

The current analyses utilized data drawn from two randomized controlled trials of continuation-phase treatment for responders to acute-phase CT for recurrent MDD (Jarrett et al., 2001; Jarrett & Thase, 2010) to evaluate the extent to which findings replicated. In these datasets, we tested the hypotheses that greater SLD predicts: (1) CT response and (2) lower depression severity for 2 years following CT response. We also tested the exploratory research question, does higher SLD mark improvement in social adjustment and patients’ CT skills? Following recommendations for testing “complex affect dynamics” such as SLD, our analyses controlled less complex patient-level symptom means and standard deviations to estimate the incremental predictive power of SLD (Dejonckheere et al., 2019).

## Method

We refer to the larger clinical-trial dataset (*N* analyzed herein = 475 of 523; Jarrett & Thase, 2010; Jarrett et al., 2013) as the primary sample and the smaller dataset (*N* analyzed herein = 146 of 156; Jarrett et al., 2001) as the replication sample. The analyzed samples were patients with sufficient data to score SLD (i.e., ≥ 3 weeks of symptom measures). Here we describe methods relevant to current analyses and refer readers to earlier publications for additional details (Jarrett et al., 2001; Jarrett et al., 2010; Jarrett et al., 2013).

### Participants

Adult outpatients provided written informed consent for assessment and treatment. The study procedures and protocol were approved by the institutional review boards at the University of Texas Southwestern Medical Center (both samples) and University of Pittsburgh Medical Center (primary sample). Participants met criteria for recurrent MDD (American Psychiatric Association, 1994, 2000) and scored ≥ 14 (primary dataset) or ≥ 16 (replication dataset) on the 17-item Hamilton Rating Scale for Depression (HRSD; Hamilton, 1960).

Diagnoses were made using the Structured Clinical Interview for *DSM-IV* (First et al., 1996) in the primary dataset or *DSM-III-R* (Spitzer et al., 1989) in the replication dataset. Participants with recurrent MDD had clear separation of major depressive episodes (both datasets) or antecedent dysthymic disorder (primary dataset). In the primary dataset, potential participants were excluded if they (a) had severe or poorly controlled concurrent medical disorders or used medication that could cause depression, (b) had psychotic or organic mental disorders, bipolar disorder, active substance dependence, or primary obsessive-compulsive or eating disorders, (c) were unable to complete questionnaires in English, (d) presented active suicide risk, (e) were < 18 or > 70 years old, (f) had previously failed to respond to ≥ 8 weeks of CT or 6 weeks of fluoxetine, or (g) were pregnant or planned to become pregnant during the first 11 months after intake. In the replication dataset, potential participants were excluded if they (a) had concurrent medical disorders that contraindicated medication or might cause depression, (b) met criteria for psychotic or organic mental disorders, borderline personality disorder, active substance dependence, or primary panic, sleep, sexual, or eating disorders, (c) were unable to complete questionnaires in English, (d) presented active suicide risk, (e) were < 18 or > 65 years old, or (f) preferred alternative treatment.

In the primary dataset (*N* = 475), 66.7% of participants were women; 4.2% were Asian, 9.3% black, 83.2% white, and 3.4% other races/ethnicities; and their mean age was 43.0 (*SD* = 11.9) years. In the replication dataset (*N* = 146), 74.7% of participants were women; 6.2% were black, 4.8% Hispanic, 87.7% white, and 1.4% other races/ethnicities; and their mean age was 41.7 (*SD* = 10.9) years.

### Procedure

#### Acute phase.

During the acute phase, patients received CT (Beck et al., 1979) in a 12week protocol. Therapists were doctoral-level clinicians who demonstrated competence in CT and received ongoing supervision. In the primary dataset, patients received 16 or 20 total CT sessions (additional sessions increased opportunity for response among patients with less early improvement). In the replication dataset, the CT protocol included 20 sessions. Sessions were 50-60 minutes each.

#### Continuation phase.

Consenting responders to acute-phase CT entered the 8-month continuation phase. In the primary sample, higher risk responders (*n* = 241, no major depressive episode and the last acute-phase HRSD score ≤ 12, but at least 1 of the last 7 acute-phase HRSD scores ≥ 7) were randomized to 8 months of continuation CT (C-CT), fluoxetine, or pill placebo with clinical management, whereas lower risk responders (*n* = 49, no major depressive episode and all of the last 7 acute-phase HRSD scores ≤ 6) were only assessed. In the replication sample, responders (*n* = 84, no major depressive episode and the last acute-phase HRSD score ≤ 9) were randomized to 8 months of C-CT or assessment-only.

In both samples, the C-CT protocol included 10 sessions (2 sessions/month for 2 months, then 1 session/month for 6 months; Jarrett, 1989; Jarrett, Vittengl & Clark, 2008). Sessions lasted about 60 minutes each.

Assessment-only in the replication sample included evaluation sessions on the same schedule as C-CT. In the primary sample, lower risk responders were assessed at 4-month intervals.

The fluoxetine and pill-placebo clinical-management protocol (Fawcett et al., 1987) was double-blinded and included only in the primary sample. Experienced pharmacotherapists provided 10 sessions on the same schedule as C-CT. The first session lasted up to 45 minutes and subsequent sessions up to 30 minutes. Pharmacotherapists evaluated symptoms and medication side effects and provided support, but they were not permitted to use the specific methods of C-CT. Research pharmacies dispensed identical active fluoxetine or placebo capsules. Doses were increased from 10 mg/day for 2 weeks, to 20 mg/day for 2 weeks, and 40 mg/day thereafter, but the dose could be decreased to lessen side effects. Modal doses of fluoxetine and placebo were 40 mg from week 8 onward. At the end of the continuation phase, study medication was tapered and stopped.

#### Follow-up phase.

In both samples, no protocol treatment was provided during follow-up. Independent evaluators assessed patients assessed every 4 months. The follow-up phase was 24 (primary sample) or 16 (replication sample) months. If patients relapsed or recurred, research personnel assisted them in finding treatment in the community.

### Measures

#### Depressive symptoms.

Clinicians completed the 17-item HRSD and patients completed the 21-item Beck Depression Inventory (BDI; Beck, Ward, Mendelson, Mock, & Erbaugh, 1961) and 30-item Inventory of Depressive Symptomatology–Self-Report (IDS-SR; Rush, Gullion, Basco, Jarrett, & Trivedi, 1996) weekly during CT. These scales have demonstrated high reliability and validity for measuring the same depressive symptom severity construct during CT (Vittengl, Clark, Kraft, & Jarrett, 2005; Vittengl, Clark, Thase, & Jarrett, 2013). Multiple measures of depressive symptoms were used to improve the reliability of assessment via aggregation across measures (e.g., Rushton et al., 1983).

#### Response to acute-phase CT.

Response was defined a priori as the absence of a major depressive episode (both samples) and an HRSD score ≤ 12 (primary sample) or ≤ 9 (replication sample) at exit from the acute phase (Jarrett et al., 2001; Jarrett et al., 2013). Assessment of MDD was conducted by an independent evaluator.

#### Social adjustment.

Patients completed the Social Adjustment Schedule—Self-report (Weissman & Bothwell, 1976) during CT week 1 and again approximately 1 week after CT. This 56-item questionnaire measures functioning in important social roles (e.g., work, leisure, parenting, marital and similar relationships). Moderately high retest stability, differentiation of depressed from non-depressed samples, and sensitivity to change in psychotherapy support the measures’ reliability and validity (Edwards et al. 1978; Weissman & Bothwell, 1976; Weissman et al. 1978). We analyzed the total score, on which higher values reflect *poorer* adjustment.

#### Cognitive therapy skills.

In the primary dataset only, patients and their therapists completed the 8-item Skills of Cognitive Therapy scale (Jarrett et al., 2011) roughly in the middle of CT and again at the end of CT. Items are rated on a 5-point frequency scale. In support of the measure’s reliability and validity as an assessment of CT-skill acquisition and use, patient and therapist ratings demonstrated moderately high retest and convergent correlations and predicted CT response (Brown et al., 2016; Jarrett et al., 2011).

#### Depression severity after CT.

Among acute-phase CT responders, independent evaluators completed the Longitudinal Interval Follow-up Evaluation (Keller et al., 1987) every 4 months after the acute phase, at study exit, and if therapists, patients, or blinded evaluators suspected a major depressive episode. From this semi-structured, retrospective interview, we analyzed psychiatric status ratings of MDD made on a 6-point scale: *1* = *no symptoms*, *2* = *one or two mild symptoms*, *3* = *obvious symptoms and moderate impairment*, *4* = *marked symptoms but does not meet full MDD criteria*, *5* = *definitely meets MDD criteria*, *6* = *meets MDD criteria with severe impairment and/or psychosis*. Supporting reliability and validity as an assessment of depression severity, MDD psychiatric status ratings have shown moderately high inter-rater and retest reliability, as well as convergence with the HRSD (Keller et al., 1987; Vittengl et al., 2014). Consistent with the interview schedule, we averaged weekly ratings into 4-month periods for analysis, with 8 periods (32 months) in the primary, and 6 periods (24 months) in the replication, samples.

### Data analytic plan and preliminary analyses

#### Scoring symptom linkage density.

We extracted items from the HRSD, BDI, and IDSSR and constructed brief scales of the nine core *DSM-5* MDD symptoms. We constructed the symptom scales based on the HRSD, BDI, and IDSR items’ face-valid item content. For example, the depressed mood scale included BDI items 1 (sadness) and 10 (crying), HRSD item 1 (depressed mood), and IDS-SR item 5 (feeling sad); and the low interest/pleasure scale included BDI items 4 (loss of enjoyment/satisfaction), 12 (loss of interest), and 21 (sexual disinterest), HRSD item 14 (loss of libido), and IDS-SR items 19 (general disinterest), 21 (anhedonia), and 22 (sexual disinterest). Online Supplement Table S1 lists the items that were used to construct each of the nine scales. Relevant item scores from the HRSD, BDI, and IDS-SR were first divided by each item’s maximum possible score (i.e., 3 for BDI and IDS-SR items; and 2 or 4 for HRSD items) to put them on the same metric and then averaged them to form the symptom scales. Symptoms were scored for 13 weeks, including weeks 1-12 in CT plus a postCT assessment approximately 1 week after CT. Online Supplement Table S2 shows descriptive statistics for the symptom scales at weeks 1 and 13. At the week-13 assessment, reliability of the symptom scales in the primary and replication datasets met or exceeded recommendations (Clark & Watson, 2019), as indexed by their average inter-item correlations (median = .55, range .24-.77; see Online Supplement Table S3).

We scored SLD for each patient as the average within-patient, time-lagged, standardized relation among the nine MDD symptoms. The time-lagged relations were derived from a series of multilevel models. In each model, at the within-patient level, we estimated

$$k_{t}=\beta_{0}+\beta_{1}k_{t-1}+\beta_{2}j_{t-1},$$

where *k* is one of nine depression symptoms observed at week *t*, and *j* is one of the other eight (*k1*) depression symptoms observed the previous week, *t-1*. The models contained only random effects to obtain patient-level relations; fixed effects (i.e., group-level relations) were not included. We computed a series of 72 models (*k* * *j* = 72), retained the *β*_2_ coefficients for each patient from each model, and averaged them to score SLD for each patient. Thus,

$$\mathrm{SLD}=\left(\Sigma \beta_{2}j_{t-1}\right)/jk,$$

and ranges from −1 to +1. For example, a positive SLD suggests that less-intense symptoms during a given week (e.g., better sleep, less guilt) predict less-intense levels of other symptoms during the subsequent week (e.g., more interest, better concentration), on average. Stated another way, SLD indexed the average symptom inter-correlation from one week to the next, across 12 one-week intervals, during CT.

Averages of 11.8 (*SD* = 2.8) and 12.2 (*SD* = 2.4) weeks of symptom data were used per patient in the primary and replication samples, respectively, to compute SLD. The median number of coefficients contributing to patients’ SLD scores was 72 (*M* = 69, range 48-72) in both the primary and replication samples. Patients’ SLD scores were roughly normally distributed in the primary (skew = −0.01, kurtosis = −1.02) and replication (skew = −0.20, kurtosis = −0.54) samples. Alpha internal consistency reliability for SLD was .95 in both samples.

For each patient, we also computed the grand mean and standard deviation across the series of nine symptom scores from CT weeks 1 through 13. We controlled these quantities in our analyses to estimate the incremental contribution of SLD when predicting outcomes.

#### Predicting outcomes from SLD.

We predicted social adjustment and CT skills at the end of acute-phase CT in linear regression, CT response in logistic regression, and depression symptom levels after acute-phase CT in repeated-measures multilevel models. All models controlled patient-level symptom means and standard deviations. Linear regression and multilevel models used maximum likelihood estimation to accommodate cases with some missing outcome data; no response data were missing in the logistic regression models. Nonsignificant (*p* > .05) two- and three-way interactions among SLD, continuation-phase arm, and time period were trimmed from the multilevel models.

## Results

### Hypothesis 1: Higher SLD predicts response to acute-phase CT.

**Figure 1.**
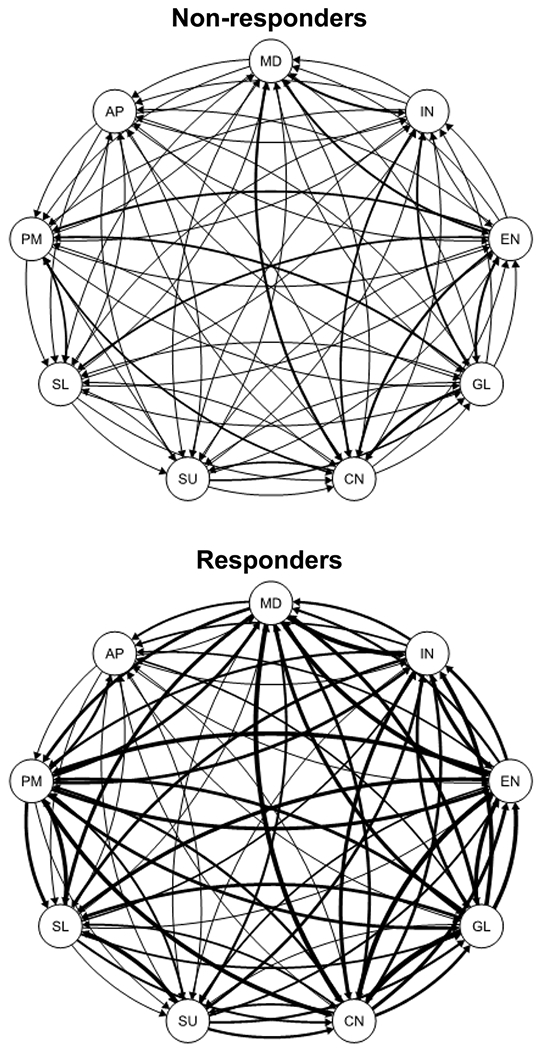
Mean within-patient, time-lagged relations among 9 depression symptoms for nonresponders (grand mean = .05) versus responders (grand mean = .12) to acute-phase cognitive therapy in the primary sample. Lines weights are proportional to the magnitude of the mean standardized regression coefficients. MD = mood, IN = interest, EN = energy, GL = guilt, CN = concentration, SU = suicidality, SL = sleep, PM = psychomotor, AP = appetite.

Table 1 provides descriptive statistics for SLD and other analyzed variables. Response to CT was defined as the absence of a major depressive episode and an HRSD score ≤ 12 (primary sample) or ≤ 9 (replication sample) at exit from the acute phase. We predicted response to CT from SLD in logistic regression models (see Table 2). Supporting the hypothesis, greater SLD predicted better odds of response in both the primary (odds ratio = 2.90) and replication (odds ratio = 3.35) samples. Figure 1 illustrates higher SLD among responders versus non-responders in the primary sample.

**Figure 2.**
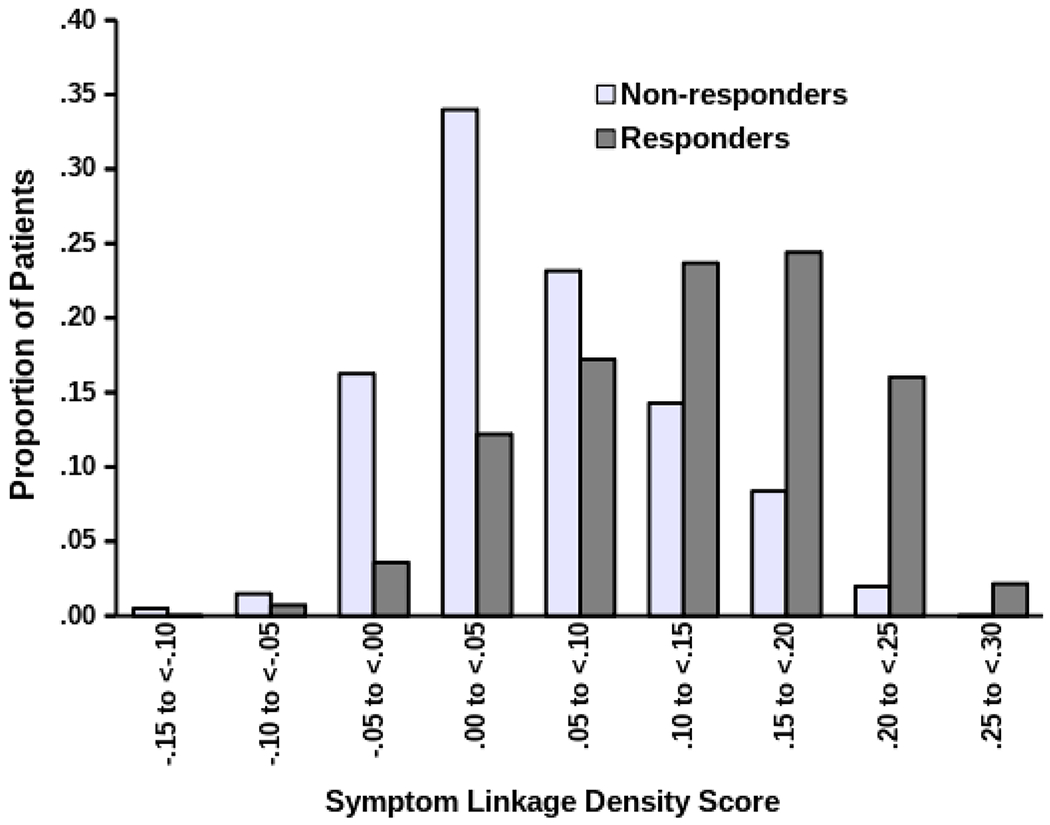
Distributions of symptom linkage density (SLD) values among responders and nonresponders, in pooled primary and replication samples

Separation of SLD distributions between responders and non-responders was considerable but incomplete. Expressed as the standardized mean difference, responders had notably higher SLD than did non-responders in the primary (*d* = 1.05) and replication (*d* = 1.33) samples. However, as shown in Figure 2, responders’ and non-responders’ SLD distributions overlapped.

### Exploratory question: Does SLD correlate with improvement in social adjustment and patient CT skills?

We predicted social adjustment at the end of CT from social adjustment at the first CT session and SLD (see Table 3). Greater SLD correlated with larger improvements in social adjustment in both the primary (standardized beta = −.20) and replication (standardized beta = −.27) samples. (Betas were negative because higher scores on the Social Adjustment Scale—Selfreport indicate *poorer* adjustment.) We also predicted patients’ CT skills at the end of CT from skills assessed mid-CT and from SLD in the primary sample. Greater SLD correlated with larger improvement in skills rated by patients (standardized beta = .19) or their therapists (standardized beta = .16).

### Hypothesis 2: Higher SLD predicts lower depressive symptoms after acute-phase CT response.

We predicted the independent evaluators’ psychiatric status ratings of MDD after CT from SLD during CT (see Table 4). In support of the hypothesis, greater SLD predicted significantly lower psychiatric status ratings for 32 months in the primary sample (standardized beta = −.11) and 24 months in the replication sample (standardized beta = −.22). Interactions of SLD with continuation-phase arm (continuation CT, fluoxetine, pill placebo, or assessment only in the primary sample; continuation CT or assessment only in the replication sample) were not significant, suggesting that acute-phase SLD predicted post-CT symptom levels roughly consistently across these continuation conditions.

## Discussion

Because MDD is defined as a multi-symptom syndrome, effective treatment necessarily involves reduction of multiple depression symptoms. Acute-phase CT is a well-established treatment for MDD, during which patients learn specific skills and improve psychosocial functioning to reduce depressive symptoms. In this context, we hypothesized that stronger connections among depressive symptoms across time during CT predict better treatment outcomes. We operationalized SLD as the mean time-lagged correlation among the nine core *DSM* symptoms of MDD across time during CT in two independent clinical-trial datasets. In support of the hypotheses, greater SLD predicted both response to CT and lower depression severity for at least 2 years after CT response. In exploratory analyses, we also found that higher SLD correlated with other possible CT mechanisms including patients’ growth in skills and social adjustment during CT. These unique findings suggest that higher SLD during CT may signal more effective treatment processes and warrants further investigation as a potential mechanism for change during CT.

We theorize that, in the context of CT for MDD, higher SLD reflects and/or facilitates growth in patients’ CT skills and psychosocial functioning. Our ideas concerning CT skills are derived from CT’s basis in learning theory, in which CT skills reflect such learning, and our ideas concerning psychosocial functioning relate to the important role of behavioral activation in efficacious CT. For example, a patient’s successful application of a CT skill to reduce a depressive symptom may be reinforced via reduction of that aversive symptom state, which increases the patient’s subsequent application of the skill or additional skills to other symptoms, leading to propagation of improvements across the symptom network, and reflected in a higher SLD score. Other patients may access this skill-symptom system by initial improvement in a symptom (e.g., restoration of hope by the choice to enter treatment) that increases acquisition and use of CT skills to reduce other symptoms. Similar hypotheses could be articulated by substituting areas of psychosocial functioning for CT skills in the preceding argument.

Future research is needed to clarify to what extent SLD reflects and/or facilitates growth in CT skills and psychosocial functioning or, alternatively, is only correlated spuriously. Research testing such mechanisms directly would require a number of design features not available in the current datasets (Kazdin, 2009). For example, comparing SLD in CT versus other active treatment (e.g., medication) and/or negative control (e.g., pill placebo) conditions would help clarify whether SLD is connected to specific features of CT, like CT skills, and/or to other mechanisms in different treatments. In addition, comparing symptom assessments before (e.g., during waitlist control) versus during CT treatment would help clarify whether SLD increases over time and whether SLD changes lag or lead patients’ improvements in symptoms, skills, and psychosocial functioning.

Acute-phase CT has well-established efficacy for reducing depressive symptoms among patients with MDD. In different contexts, SLD may reflect non-therapeutic processes and predict different outcomes. For example, among persons with untreated MDD, greater SLD may imply that a problem in one area (e.g., poor sleep) has spread to other areas (e.g., trouble concentrating). Such cascade effects may produce poorer outcomes in persons with untreated MDD (cf. Selby et al., 2016; Murray et al, 2020). Consistent with this possibility, persons with MDD (treatment status not reported) showed stronger time-lagged connections among negative emotions, compared to normal controls in a previous study (Pe et al., 2015). This contrasting finding adds context to our current results showing higher SLD among CT responders versus non-responders.

We also theorize that SLD marks individual differences in the coherence of the depression syndrome. That is, some people may have “tighter” or “looser” constellations of depressive symptoms. Symptoms that form a coherent syndrome may present a clearer treatment target (Cuthbert, 2014). Similarly, classification of psychopathology, including identifying overlapping and unique categories or dimensions, is an active area of research (Krueger et al., 2018). At the individual-patient level, SLD may be relevant to such questions as, “does a specific list of symptoms delineate a single psychopathological entity…?” (Krueger et al., 2018, p. 282). More specifically, it may be more or less appropriate to diagnose individuals with MDD depending on the strength of their symptom interconnections. Future research on SLD incorporating a wider range of symptoms (e.g., from anxiety and personality disorder) might clarify at an individual-patient level whether MDD forms a distinct syndrome and treatment target. Thus, high SLD may mark a coherent MDD syndrome more amenable to depressiontargeted treatments, as reflected in lower depressive symptomatology for 2 years after CT response in the current analyses.

Our current analyses included several features to help rule out artifactual or spurious findings. First, our results replicated across samples (CT response, post-CT depression severity, social adjustment) and raters (CT skills). Second, we controlled symptom means and standard deviations to establish the incremental validity of SLD. In contrast, in some past studies predicting well-being from measures of emotion dynamics, more-complex indices broadly similar to SLD have not shown incremental predictive power. Finally, the majority of our outcome measures (CT skills, social adjustment, post-CT depression severity) had no overlap with the data used to compute SLD, and SLD distributions were distinct but overlapping between acute-phase CT responders and non-responders. Consequently, our definition and measurement of SLD offered information beyond symptom levels and changes.

The current study also has notable limitations. First, participants had carefully diagnosed, recurrent MDD and were treated by proficient cognitive therapists in a research protocol. Similarly, the majority of participants were white and female. Generalization to other patient populations and treatments is uncertain. Evaluation of SLD in the context of other acute-phase treatments (e.g., pharmacotherapy) and untreated groups would be particularly valuable in clarifying the predictive value of the SLD construct. Second, depressive symptoms were measured with three well-established instruments (HRSD, BDI, IDS-SR) to form MDD symptom scales, but other instruments and symptom measures might yield different results. Moreover, potential ambiguities in the *DSM-5* criteria for a major depressive episode (e.g., both insomnia and hypersomnia are included in criterion 4) were duplicated in our symptom scales, possibly limited the validity of SLD estimates, and could be addressed in future research using symptom inventories with psychometrically “purer” scales (e.g., Watson et al., 2012). Third, effect sizes for the incremental relations of SLD with social adjustment, CT skills, response, and post-CT depression severity were small-to-moderate, highlighting that other processes are also important in CT patients’ outcomes. For example, the potential relations of comorbidity (e.g., anxiety and personality disorder) and depression subtypes (e.g., single-episode vs. recurrent, atypical) with SLD are unknown and a potentially important topic for future research. Fourth, the clinical trials were not designed for assessment of SLD and sample sizes were limited, particularly after acute-phase CT. Finally, replication of SLD as a treatment outcome predictor in other datasets is needed before clinical application, and application in routine clinical practice may necessitate development and validation of a simpler SLD formula or heuristic. Similarly, our exploratory analyses concerning SLD’s relations to other potential CT processes (i.e., improvement in psychosocial functioning and patient skills) require explication in future research.

Investigation of SLD may offer new insights into the patients and processes involved in CT’s well-established efficacy for MDD. Given poor replication of some important findings documented in psychological science (Open Science Collaboration, 2015), our findings that SLD predicted patient outcomes in two large, independent clinical trials with longitudinal follow-up are important strengths. We hypothesize that greater SLD, at least in part, reflects patients’ generalization of CT skills and psychosocial functioning improvements. We also speculate that SLD has the potential to clarify other important issues, such as the coherence of the depression syndrome for individual patients. We hope to encourage additional research on this potentially valuable construct and hypotheses.

## Supplementary Material

1745109_sup_info.

## Figures and Tables

**Table 1:** Descriptive Statistics for Variables during and after Acute-phase Cognitive Therapy (CT)

Variable	*n*	*M* or %	*SD*
Primary Sampl			
During CT			
Symptom mean	475	0.26	0.11
Symptom standard deviation	475	0.20	0.04
Symptom linkage density	475	0.10	0.08
Patient-rated CT skills mid-CT	398	3.33	0.66
Patient-rated CT skills at the end of CT	365	3.58	0.72
Therapist-rated CT skills mid-CT	416	3.21	0.70
Therapist-rated CT skills at the end of CT	383	3.42	0.80
Social adjustment at the start of CT	447	2.53	0.44
Social adjustment at the end of CT	352	1.97	0.43
Responded to CT	475	68.0%	
Responders after CT			
MDD PSR months 1-4	245	1.98	0.92
MDD PSR months 5-8	235	1.88	0.90
MDD PSR months 9-12	216	1.82	0.97
MDD PSR months 13-16	197	1.70	0.91
MDD PSR months 17-20	188	1.75	1.06
MDD PSR months 21-24	175	1.77	1.02
MDD PSR months 25-28	166	1.71	1.03
MDD PSR months 29-32	159	1.57	0.90
Replication Sample			
During CT			
Symptom mean	146	0.23	0.11
Symptom standard deviation	146	0.20	0.04
Symptom linkage density	146	0.13	0.08
Social adjustment at the start of CT	145	2.52	0.41
Social adjustment at the end of CT	126	1.82	0.40
Responded to CT	146	65.1%	
Responders after CT			
MDD PSR months 1-4	79	1.33	0.78
MDD PSR months 5-8	77	1.49	1.10
MDD PSR months 9-12	72	1.43	0.92
MDD PSR months 13-16	69	1.46	1.03
MDD PSR months 17-20	67	1.44	0.94
MDD PSR months 21-24	64	1.39	0.99

*Note*. MDD PSR = average major depressive disorder psychiatric status ratings (higher scores indicate more severe depression).

**Table 2:** Prediction of Response to Acute-phase Cognitive Therapy

Predictor	*B*	*SE*	*p*	Odds Ratio
Primary Sample				
Intercept	3.47	0.82		
Symptom mean	−21.60	2.51	<.001	0.08
Symptom standard deviation	9.81	5.04	.052	1.50
Symptom linkage density	14.11	2.25	<.001	2.90
Replication Sample				
Intercept	−0.26	1.66		
Symptom mean	−23.49	4.87	<.001	0.10
Symptom standard deviation	24.00	10.64	.024	2.50
Symptom linkage density	14.81	3.85	<.001	3.35

*Note*. Tabled values are from logistic regression models. Odds ratios scaled to reflect 1 *SD* differences in the predictors.

**Table 3:** Prediction of Social Adjustment and Patients’ Skills at the End of Acute-phase Cognitive Therapy (CT)

Predictor	*B*	*SE*	*p*	*β*
Social Adjustment: Primary Sample				
Intercept	1.08	0.10		
Social adjustment at the start of CT	0.39	0.04	<.001	.37
Symptom mean	2.15	0.21	<.001	.53
Symptom standard deviation	−2.03	0.52	<.001	−.18
Symptom linkage density	−1.73	0.23	<.001	−.28
Social Adjustment: Replication Sample				
Intercept	1.24	0.18		
Social adjustment at the start of CT	0.12	0.07	.102	.11
Symptom mean	2.17	0.32	<.001	.55
Symptom standard deviation	0.06	0.89	.944	.01
Symptom linkage density	−1.43	0.34	<.001	−.28
Patient-rated Skills: Primary Sample				
Intercept	1.08	0.20		
Skills mid-CT	0.65	0.04	<.001	.58
Symptom mean	−1.38	0.36	<.001	−.21
Symptom standard deviation	2.19	0.91	.017	.12
Symptom linkage density	1.92	0.38	<.001	.19
Therapist-rated Skills: Primary Sample				
Intercept	0.87	0.21		
Skills mid-CT	0.77	0.04	<.001	.66
Symptom mean	−0.85	0.36	.018	−.12
Symptom standard deviation	0.41	0.92	.652	.02
Symptom linkage density	1.75	0.40	<.001	.16

*Note*. Tabled values are from linear regression models. Higher scores on the Social Adjustment Scale—Self-report indicate *poorer* adjustment.

**Table 4:** Prediction of Depression Severity after Response to Acute-phase Cognitive Therapy (CT)

Predictor	*df*	*F*	*p*	*β*
Primary Sample				
Continuation-phase arm	3, 238	0.27	.850	
Time period	7, 238	3.32	.002	
Arm * time period	21, 238	1.92	.011	
Symptom mean	1, 238	27.06	<.001	.35
Symptom standard deviation	1, 238	3.65	.057	−.12
Symptom linkage density	1, 238	5.22	.023	−.11
Replication Sample				
Continuation-phase arm	1, 79	2.20	.143	
Time period	5, 79	1.19	.324	
Symptom mean	1, 79	3.66	.060	.16
Symptom standard deviation	1, 79	1.06	.307	.09
Symptom linkage density	1, 79	12.10	<.001	−.22

*Note*. Tabled values are from repeated-measures multilevel model used to predict psychiatric status ratings of major depressive disorder after CT response. In the primary sample, 8-month continuation-phase arms included continuation cognitive therapy, fluoxetine, or pill placebo with clinical management for higher-risk responders or assessment-only for lower-risk responders. In the replication sample, 8-month continuation -phase arms included continuation cognitive therapy or assessment-only. Time period refers to four-month blocks through 32 months (i.e., 8 periods) in the primary sample or 24 months (i.e., 6 periods) in the replication sample. Nonsignificant (*p* > .05) two- and three-way interactions among continuation-phase arm, time period, and symptom linkage density were tested and trimmed from the final models shown.
