# Supplementary material for: Does Symptom Linkage Density Predict Outcomes in Cognitive Therapy for Recurrent Depression?
Source: J Psychopathol Behav Assess. Author manuscript; Available in PMC 2023 Jun 1. (PMC9354858; doi:10.1007/s10862-021-09914-y)
Supplement: 1745109_sup_info. [file NIHMS1745109-supplement-1745109_sup_info_.doc]

Does Symptom Linkage Density Predict Outcomes

in Cognitive Therapy for Recurrent Depression?

Online Supplement

Table S1

*Scoring Nine DSM-5 Symptoms of Major Depressive Disorder*

This document lists depressive symptom measure items and how they map onto the 9 core *DSM-5* symptoms for MDD. The scoring focused on the 21-item BDI, 17-item HRSD, and 30-item IDS-SR, and excluded additional items that are sometimes part of the BDI and HRSD. The applicable items were standardized and averaged to form each symptom scale.

BDI

1-mood (included on depressed mood scale)

2-hopelessness (excluded, not among core *DSM-5* criteria)

3-self-criticism/guilt (included on guilt scale)

4-interest/pleasure (included on interest scale)

5-guilt (included on guilt scale)

6-guilt (included on guilt scale)

7-self-criticism/guilt (included on guilt scale)

8-self-criticism/guilt (included on guilt scale)

9- hopelessness/suicide (included on suicidality scale)

10-mood (included on depressed mood scale)

11-irritable mood (excluded, not clearly among core *DSM-5* criteria for adults)

12-interest/pleasure (included on interest scale)

13-decision/concentration (included on concentration scale)

14-self-criticism/guilt (excluded, item about appearance specifically rather than *DSM-5* guilt criterion)

15-energy/fatigue-work (excluded, item splits conceptually between energy and interest criteria)

16-sleep (included in sleep scale)

17-fatigue (included in energy scale)

18-appetite/weight change (included in appetite/weight scale)

19-appetite/weight change (included in appetite/weight scale)

20-somatic concerns (excluded, not among core *DSM-5* criteria)

21-interest/pleasure (included on interest scale)

HRSD

1-mood (included on depressed mood scale)

2-guilt (included on guilt scale)

3-suicide (included on suicidality scale)

4-sleep (included in sleep scale)

5-sleep (included in sleep scale)

6-sleep (included in sleep scale)

7-interest/energy in work (excluded, item splits conceptually between energy and interest criteria)

8-psychomotor change (included on psychomotor scale)

9-psychomotor change (included on psychomotor scale)

10-anxiety (excluded, not among core *DSM-5* criteria)

11-anxiety (excluded, not among core *DSM-5* criteria)

12-appetite (included in appetite/weight scale)

13-energy (included in energy scale)

14-interest (included on interest scale)

15-somatic (excluded, not among core *DSM-5* criteria)

16-insight (excluded, not among core *DSM-5* criteria)

17-weight change (included in appetite/weight scale)

IDS-SR

1-early insomnia (included in sleep scale)

2-middle insomnia (included in sleep scale)

3-late insomnia (included in sleep scale)

4-hypersomnia (included in sleep scale)

5-sad (included on depressed mood scale)

6-irritable (excluded, not among *DSM-5* criteria for adults)

7-anxious (excluded, not among core *DSM-5* criteria)

8-reactive mood (excluded, not among core *DSM-5* criteria)

9-time of day (excluded, not among core *DSM-5* criteria)

10-mood quality/grief (excluded, not among core *DSM-5* criteria)

11-decreased appetite (included in appetite/weight scale)^a^

12-increased appetite (included in appetite/weight scale)^a^

13-decreased weight (included in appetite/weight scale)^a^

14-increased weight (included in appetite/weight scale)^a^

15-concentration (included on concentration scale)

16-self-view (included on guilt scale)

17-hopelessness (excluded, not among core *DSM-5* criteria)

18-death/suicide (included on suicidality scale)

19-interest (included on interest scale)

20-energy (included in energy scale)

21-pleasure (included on interest scale)

22-interest in sex (included on interest scale)

23-slowed down (included on psychomotor scale)

24-restless/agitated (included on psychomotor scale)

25-aches/pains (excluded, not among core *DSM-5* criteria)

26-somatic (excluded, not among core *DSM-5* criteria)

27-panic (excluded, not among core *DSM-5* criteria)

28-GI symptoms (excluded, not among core *DSM-5* criteria)

29-interpersonal sensitivity (excluded, not among core *DSM-5* criteria)

30-leaden paralysis (excluded, not among core *DSM-5* criteria)

^a^ Consistent with the scoring of the IDS-SR, the maximum of items 11 and 12, and the maximum of items of 13 and 14, were retained for scoring the appetite scale.

Table S2

*Mean Scores on Nine DSM-5 Symptoms for Major Depressive Disorder*

|  | Week 1 | | | Week 13 | | |
| --- | --- | --- | --- | --- | --- | --- |
| Symptom scale | *N* | *M* | *SD* | *N* | *M* | *SD* |
| Primary Sample | | | | | | |
| Mood | 475 | 0.54 | 0.19 | 395 | 0.20 | 0.21 |
| Interest | 475 | 0.50 | 0.21 | 395 | 0.24 | 0.24 |
| Energy | 475 | 0.60 | 0.23 | 395 | 0.26 | 0.25 |
| Guilt | 475 | 0.45 | 0.20 | 395 | 0.16 | 0.18 |
| Concentration | 468 | 0.53 | 0.20 | 368 | 0.21 | 0.22 |
| Suicidality | 475 | 0.20 | 0.19 | 395 | 0.06 | 0.13 |
| Sleep | 475 | 0.43 | 0.23 | 395 | 0.24 | 0.21 |
| Psychomotor | 475 | 0.27 | 0.14 | 395 | 0.09 | 0.11 |
| Appetite | 475 | 0.21 | 0.17 | 395 | 0.08 | 0.12 |
| Replication Sample | | | | | | |
| Mood | 146 | 0.53 | 0.17 | 128 | 0.16 | 0.20 |
| Interest | 146 | 0.51 | 0.21 | 128 | 0.21 | 0.22 |
| Energy | 146 | 0.63 | 0.20 | 128 | 0.21 | 0.25 |
| Guilt | 146 | 0.43 | 0.22 | 128 | 0.12 | 0.15 |
| Concentration | 145 | 0.57 | 0.18 | 126 | 0.18 | 0.22 |
| Suicidality | 146 | 0.20 | 0.18 | 128 | 0.05 | 0.12 |
| Sleep | 146 | 0.44 | 0.21 | 128 | 0.23 | 0.21 |
| Psychomotor | 146 | 0.25 | 0.14 | 128 | 0.07 | 0.11 |
| Appetite | 146 | 0.21 | 0.15 | 128 | 0.07 | 0.11 |

*Note*. Data shown only for patients with symptom linkage density scores.

Table S3

*Reliability Estimates for Depression Symptom Scales*

| Symptom scale | Average inter-item correlation | Cronbach’s alpha coefficient |
| --- | --- | --- |
| Primary Sample | | |
| Mood | .57 | .84 |
| Interest | .55 | .90 |
| Energy | .66 | .86 |
| Guilt | .54 | .89 |
| Concentration | .73 | .84 |
| Suicidality | .69 | .87 |
| Sleep | .32 | .79 |
| Psychomotor | .34 | .67 |
| Appetite | .24 | .68 |
| Replication Sample | | |
| Mood | .66 | .88 |
| Interest | .55 | .90 |
| Energy | .77 | .91 |
| Guilt | .55 | .89 |
| Concentration | .77 | .87 |
| Suicidality | .66 | .85 |
| Sleep | .29 | .76 |
| Psychomotor | .39 | .72 |
| Appetite | .27 | .72 |

*Note*. Estimates computed using week 13 observations.
